# Profound Interfacial Effects in CoFe_2_O_4_/Fe_3_O_4_ and Fe_3_O_4_/CoFe_2_O_4_ Core/Shell Nanoparticles

**DOI:** 10.1186/s11671-018-2481-x

**Published:** 2018-03-01

**Authors:** Dmytro Polishchuk, Natalia Nedelko, Sergii Solopan, Anna Ślawska-Waniewska, Vladyslav Zamorskyi, Alexandr Tovstolytkin, Anatolii Belous

**Affiliations:** 1 Institute of Magnetism of the NAS of Ukraine and MES of Ukraine, 36b Vernadsky Blvd., Kyiv, 03142 Ukraine; 2 Institute of Physics, Polish Academy of Sciences, Aleja Lotnikow 32/46, PL-02668 Warsaw, Poland; 3 V.I. Vernadskii Institute of General and Inorganic Chemistry of the NAS of Ukraine, 32/34 Palladina Ave., Kyiv, 03680 Ukraine; 4 Faculty of Radiophysics, Electronics and Computer Systems, Taras Shevchenko National University of Kyiv, 4G Glushkova Ave., Kyiv, 03680 Ukraine

**Keywords:** Spinel ferrites, Core/shell nanoparticles, Magnetic properties, Effective anisotropy, Interfacial effects, 81.07.Bc, 75.50.Tt, 75.30.Gw

## Abstract

Two sets of core/shell magnetic nanoparticles, CoFe_2_O_4_/Fe_3_O_4_ and Fe_3_O_4_/CoFe_2_O_4_, with a fixed diameter of the core (~ 4.1 and ~ 6.3 nm for the former and latter sets, respectively) and thickness of shells up to 2.5 nm were synthesized from metal chlorides in a diethylene glycol solution. The nanoparticles were characterized by X-ray diffraction, transmission electron microscopy, and magnetic measurements. The analysis of the results of magnetic measurements shows that coating of magnetic nanoparticles with the shells results in two simultaneous effects: first, it modifies the parameters of the core-shell interface, and second, it makes the particles acquire combined features of the core and the shell. The first effect becomes especially prominent when the parameters of core and shell strongly differ from each other. The results obtained are useful for optimizing and tailoring the parameters of core/shell spinel ferrite magnetic nanoparticles for their use in various technological and biomedical applications.

## Background

Core/shell architecture has acquired increasing interest due to the possibility of combining different materials and fabricating nanostructures with improved characteristics [1, 2]. In addition to varying size, shape, and composition, tuning of magnetic properties through the interface coupling of different magnetic materials becomes a prevailing strategy, introducing a new variable for the rational material design and property control in fundamental science and technological applications [3, 4]. Recent studies have demonstrated some merits of bimagnetic core/shell nanocrystals in improving the energy product of permanent magnets [5], enhancing the thermal stability of magnetic nanocrystals to overcome the “superparamagnetic limitation” in recording media [6], and optimizing the parameters of nanoparticles for biomedical applications [3, 7]. The exploration of core/shell combinations of different magnetic materials will provide a better fundamental understanding of magnetic interactions and make it possible to achieve the desirable magnetic characteristics for various specific applications.

As one of the most important and widely utilized magnetic materials, the spinel ferrite system consists of both magnetically hard and soft materials. For example, cobalt ferrite (CoFe_2_O_4_) is magnetically hard with a large magnetocrystalline anisotropy constant *K* > 10^6^ erg/cm^3^ [5, 6]. On the other hand, magnetite (Fe_3_O_4_) is a ferrite with a much smaller magnetic anisotropy constant *K* ∼ (10^4^ ÷ 10^5^) erg/cm^3^ [8, 9]. Due to the same crystallographic structure and almost negligible lattice mismatch among these spinel ferrites, it should be markedly controllable to epitaxially grow a uniformed shell over a core. Among other things, such kind of the well-defined bimagnetic spinel ferrite nanocrystals with core/shell architecture can provide a better platform for the fundamental understanding of magnetism and the relationship between the crystalline structure, the morphology, and the physical properties.

According to the data of the recent review paper [10], the magnetic properties of core/shell structures are determined by such parameters as a size, particular order (soft/hard or hard/soft), and geometric shape of core and shell (spherical or planar). In addition, the magnetic properties depend on the difference in magnetic parameters between the core and shell materials as well as on the presence or absence of the dipolar and exchange-coupled interactions that affect the spin reversal processes [11]. The no less important factors in determining the magnetic properties of the core/shell structures are their size distribution and microstructure change when processed at high temperatures. Core and shell may coalesce at high temperatures forming a structure of core nanoparticles embedded in a shell matrix [12]. Due to these obstacles, a number of issues related to understanding the surface and interface phenomena, the mechanisms of magnetic coupling at the core-shell interface and others, remain to be explored.

The majority of the publications on core/shell magnetic nanoparticles (MNPs) deal with the co-precipitation of slightly soluble compounds from aqueous solutions [13–15]. The complex and uncontrollable mechanism of such reactions involves crystal nucleation, growth, coarsening, or agglomeration processes, which occur simultaneously. This often results in the agglomeration of nanoparticles. In the works of [16, 17], MFe_2_O_4_ nanoparticles (M = Mn, Fe, Co, Ni, Zn) with spinel structure were synthesized from metal chlorides in a diethylene glycol (DEG) solution. The complex reaction of DEG with transition metal cations makes it possible to separate in time the crystal nucleation and growth processes and, thus, to partially control the particles’ size and aggregation. It looks appealing to use these advantages to clarify some of the issues mentioned above.

In light of the above comments, the aims of the present work were to synthesize CoFe_2_O_4_/Fe_3_O_4_ and Fe_3_O_4_/CoFe_2_O_4_ core/shell nanoparticles from a DEG solution, understand the effect of core/shell architecture on magnetization and effective anisotropy of MNPs, and pave the way to fabricate MNPs with tunable magnetic parameters for various technological and biomedical applications.

## Experimental

### Details of Synthesis

For the synthesis of CoFe_2_O_4_/Fe_3_O_4_ and Fe_3_O_4_/CoFe_2_O_4_ core/shell MNPs, iron (III) chloride nonahydrate (97% FeCl_3_·9H_2_O, Sigma Aldrich), cobalt (II) nitrate hexahydrate (98% Co(NO_3_)_2_·6H_2_O, Sigma Aldrich), iron (II) sulfate heptahydrate (99% FeSO_4_·7H_2_O, Sigma Aldrich), sodium hydroxide (98% NaOH), and diethylene glycol (99% DEG, Sigma Aldrich) were used as starting reagents. All stages of synthesis were carried out in a three-neck flask in argon atmosphere according to the method described in Reference [18]. At the first stage of the synthesis, individual CoFe_2_O_4_ and Fe_3_O_4_ MNPs were prepared, which were subsequently used as respective cores of CoFe_2_O_4_/Fe_3_O_4_ and Fe_3_O_4_/CoFe_2_O_4_ core/shell MNPs.

#### Synthesis of CoFe_2_O_4_ MNPs

Co(NO_3_)_2_⋅6H_2_O and FeCl_3_⋅9H_2_O in a molar ratio (1:2) were dissolved in DEG. At the same time, NaOH in DEG was prepared. The alkali solution was added to the mixture of Co(NO_3_)_2_·6H_2_O and FeCl_3_·9H_2_O salts, and the resulting mixture was stirred for 2 h. The obtained solution was heat-treated at 200–220 °C (60 min). Oleic acid was then added to the DEG solution, and the mixture was further stirred for 10–20 min. The resulting colloidal solution after cooling was centrifuged, redispersed in ethanol, and dried in the air.

#### Synthesis of Fe_3_O_4_ MNPs

FeSO_4_·7H_2_O and FeCl_3_·9H_2_O in a molar ratio (1:2) were dissolved in DEG. At the same time, NaOH in DEG was prepared. The alkali solution was added to the mixture of the salts FeSO_4_·7H_2_O and FeCl_3_·9H_2_O, and the resulting mixture was stirred for 2 h. The obtained solution was heat-treated at 200–220 °C (60 min). Oleic acid was then added to the diethylene glycol solution, and the mixture was further stirred for 10–20 min. The resulting precipitate after cooling was centrifuged, redispersed in ethanol, and dried in the air.

#### Synthesis of CoFe_2_O_4_/Fe_3_O_4_ MNPs

**Fig. 1:**
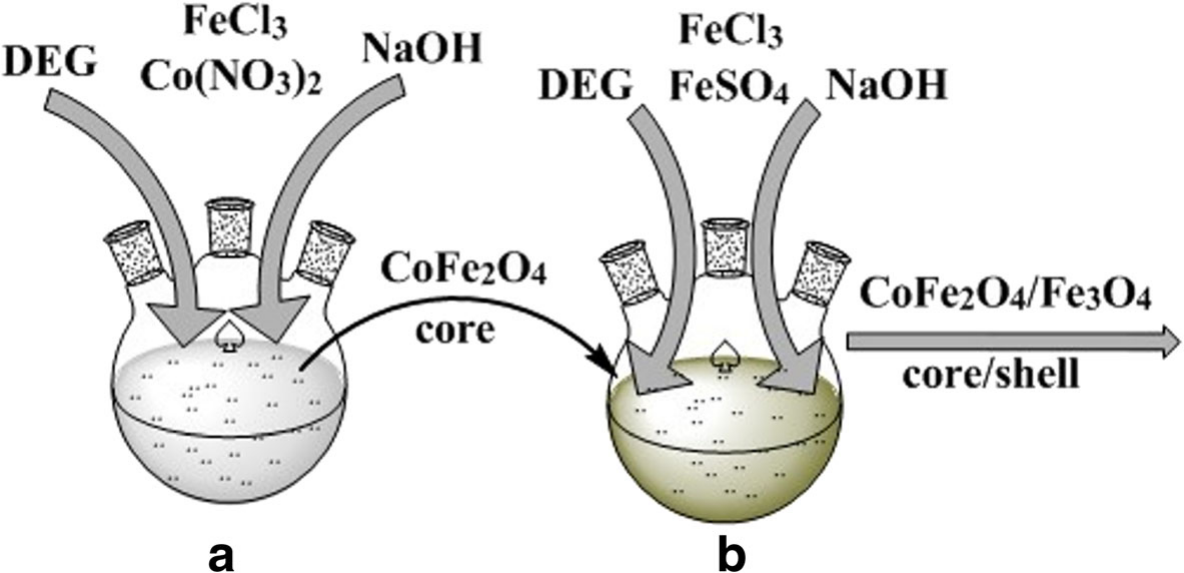
Scheme of the synthesis of CoFe_2_O_4_/Fe_3_O_4_ core/shell nanoparticles: synthesis of CoFe_2_O_4_ core at the first stage (**a**) and final product at the second stage (**b**)

CoFe_2_O_4_/Fe_3_O_4_ nanoparticles with a core/shell structure were synthesized in a three-neck flask in the argon atmosphere. As a core of the MNPs, CoFe_2_O_4_ nanoparticles, which were synthesized by the method described above, were used. The average size of CoFe_2_O_4_ core was ~ 4.1 nm. At the first stage, the necessary amount of pre-synthesized CoFe_2_O_4_ nanoparticles was set apart (Fig. 1a). At the second stage, the starting solution for the synthesis of Fe_3_O_4_ shell was prepared—FeSO_4_·7H_2_O and FeCl_3_·9H_2_O were taken in a stoichiometric ratio of 1:2 and mixed with DEG (Fig. 1b). NaOH in DEG was added dropwise to the obtained solution and stirred for 1 h. The pre-synthesized core (CoFe_2_O_4_) nanoparticles were added to the obtained reaction mixture, and the resulting product was mixed for 1 h under the action of ultrasound. The obtained reaction mixture was heated up to 200 °C with a rate of 2–3 °C/min and maintained at this temperature for 1.5 h. The precipitate was separated by centrifugation and dried in the air or kept in the hexane solution.

The amount of shell material to be precipitated on the core was calculated as follows. First, the volume of the shell material per one core/shell particle, *V*_shell_, was calculated by the formula: *V*_shell_ = 4/3*π*[(*R*_2_)^3^−(*R*_1_)^3^], where *R*_1_ and *R*_2_ are the radii of the initial and coated spherical particle, respectively. Then the mass of the shell material per one particle, *m*_shell_, was found as *m*_shell_ = *ρ*·*V*_shell_, where *ρ* is the shell density (5 g/sm^3^). Accordingly, the mass of the core material per one particle, *m*_core_, was calculated. The knowledge of *m*_shell_/*m*_core_ ratio made it possible to find the mass of the shell material for any chosen mass of the core material. For example, to cover 1 g of CoFe_2_O_4_ nanoparticles with an average size of 4.1 nm with a shell of about 1 nm, it requires 1.2 g of Fe_3_O_4_.

#### Synthesis of Fe_3_O_4_/CoFe_2_O_4_ MNPs

Fe_3_O_4_/CoFe_2_O_4_ nanoparticles with a core/shell structure were synthesized in a three-neck flask in the argon atmosphere. As a core of the MNPs, Fe_3_O_4_ nanoparticles, which were synthesized by the method described above, were used. The average size of Fe_3_O_4_ core was ~ 6.3 nm. At the first stage, the necessary amount of pre-synthesized Fe_3_O_4_ nanoparticles was set apart. At the second stage, the starting solution for the synthesis of CoFe_2_O_4_ shell was prepared—Co(NO_3_)_2_·6H_2_O and FeCl_3_·9H_2_O were dissolved in DEG, and the solution was stirred for 10–20 min. NaOH in DEG was added dropwise to the resulting solution and stirred for 1 h. Then the pre-synthesized core (Fe_3_O_4_) nanoparticles were added to the obtained reaction mixture, and the resulting product was mixed for 1 h under the action of ultrasound. The obtained reaction mixture was heated up to 200 °C with a rate of 2–3 °C/min and maintained at this temperature for 1.5 h. The precipitate was separated by centrifugation and dried in the air or kept in the hexane solution.

The amount of shell (CoFe_2_O_4_), which was precipitated on the core (Fe_3_O_4_), was calculated by the technique described above, taking into account that the initial average size of core nanoparticles was 6.3 nm.

According to the methods described above, two sets of core/shell MNPs were synthesized. The first one includes MNPs with CoFe_2_O_4_ core and Fe_3_O_4_ shell with the calculated effective thickness of the shell 0, 0.05, 1, and 2.5 nm. The second set includes MNPs with Fe_3_O_4_ core and CoFe_2_O_4_ shell with the calculated effective thickness of the shell 0, 0.05, and 1 nm. In the text below, the first and second sets will be denoted as Co/Fe(*t*_Fe_) and Fe/Co(*t*_Co_), respectively.

### Details of Characterization and Measurements

Nanostructured powders were investigated by PANalytical’s X-ray diffraction (XRD) system on X’Pert powder diffractometer (Co-K*α* radiation, voltage 45 kV, current 35 mA, Ni filter). Calculations of the intensity redistribution and angles of X-ray peaks for individual compounds and core/shell nanoparticles were performed by PeakFit 4.12 software using individual peaks with maximum intensity in the range of 2*θ* angles from 38° to 46°.

The size and morphology of powder particles have been determined by means of a JEM-1230 scanning electron microscope. To calculate the particle size distribution, TEM images were analyzed according to the procedure described by Peddis et al. [19].

Magnetic measurements were performed in the 5–350 K temperature range using a commercial Quantum Design Physical Property Measurement System (PPMS) equipped with vibrating sample magnetometer. Magnetic moment was measured upon heating for both zero-field-cooled (ZFC) and field-cooled (FC) conditions. Isothermal magnetic hysteresis loops were measured at 5 and 300 K in magnetic fields from − 60 to 60 kOe.

## Results

### XRD and TEM Investigations

**Fig. 2:**
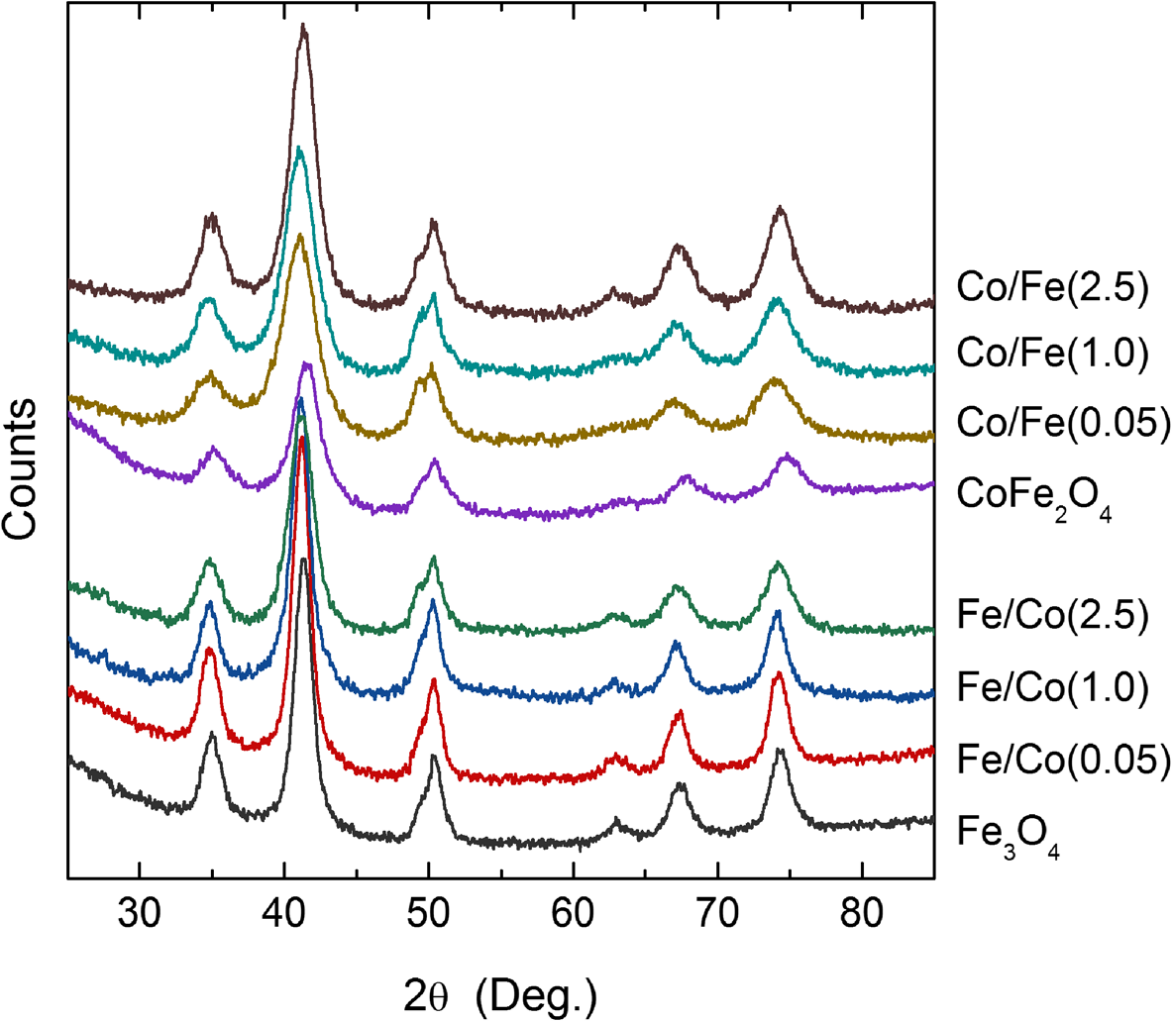
XRD patterns for the nanoparticles under study

XRD patterns for the nanoparticles under study indicate that all synthesized samples have a cubic spinel structure (JCPDS card number 19-0629 [20]). No traces of impurity phases have been revealed (Fig. 2).

Taking into account that core and shell have the same density, they cannot be distinguished by the contrast of the TEM image. Therefore, to confirm the formation of core/shell structure, we used a comparative analysis of XRD patterns collected from separate CoFe_2_O_4_ and Fe_3_O_4_ MNPs, mechanical mixture composed of these compounds taken in 1:1 ratio, and supposed core/shell structures. As described in detail in Reference [18], the results confirm the formation of core/shell structure rather than a mechanical mixture.

**Fig. 3:**
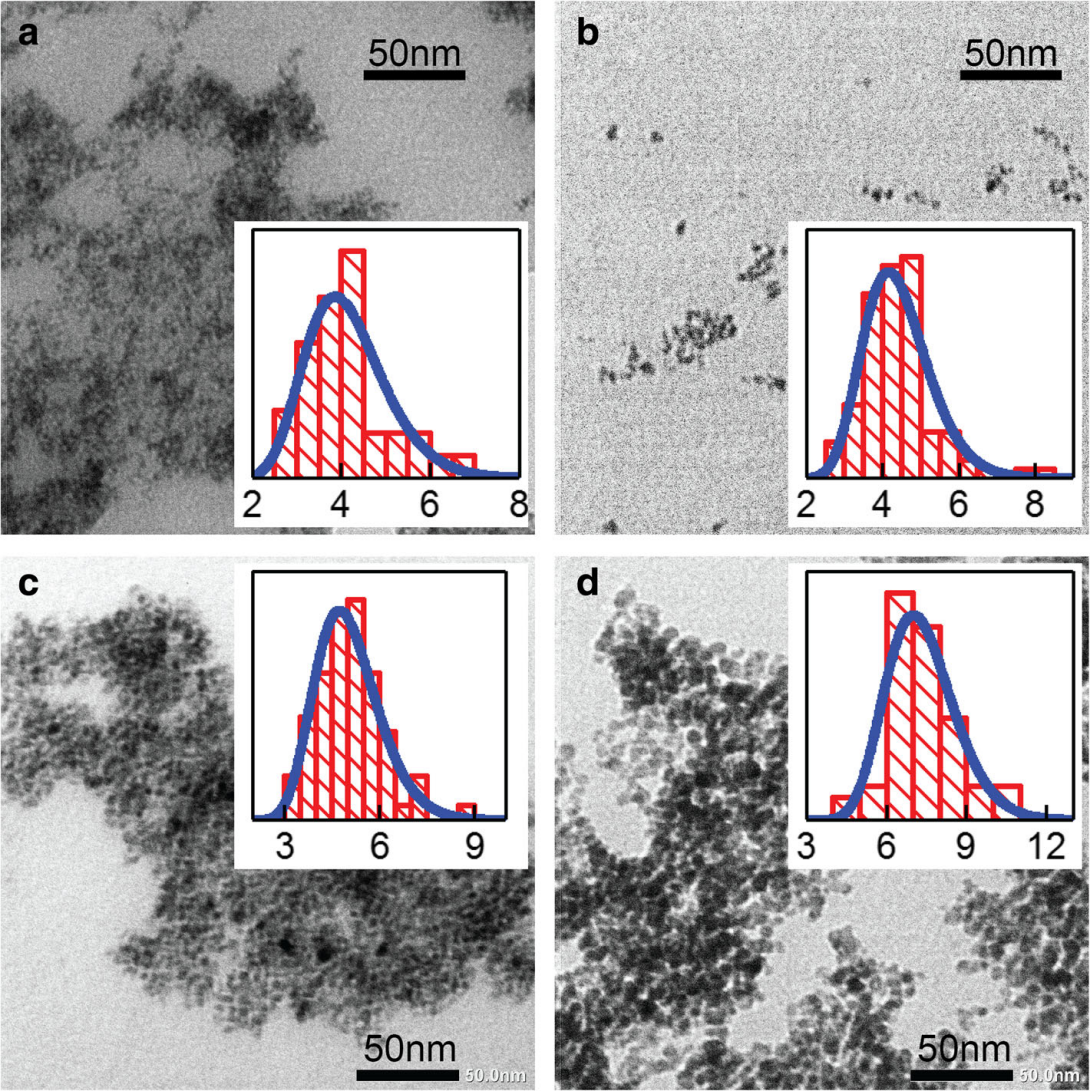
TEM images of Co/Fe(*t*_Fe_) nanoparticles with *t*_Fe_ = 0 nm (**a**), 0.05 nm (**b**), 1 nm (**c**), and 2.5 nm (**d**). Insets show the diagrams of size distribution for corresponding ensembles of nanoparticles (the units of abscissa axes are nanometers)

As can be estimated from the results of TEM investigations, the size of the Co/Fe(*t*_Fe_) сore/shell nanoparticles increases from ~ 4.1 to ~ 7.3 nm with the increase of calculated *t*_Fe_ from 0.05 to 2.5 nm (Fig. 3). It should be noted that experimentally obtained shell thicknesses are smaller than calculated ones. This can be explained by the fact that not all amount of shell material precipitated on the surface of the core. It is also noteworthy that for the case where calculated shell thickness is 0.05 nm, the particles have island-like shell rather than continuous one, since the thickness of the shell cannot be smaller than the lattice parameter of Fe_3_O_4_.

**Fig. 4:**
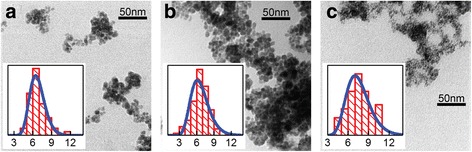
TEM images of Fe/Co(*t*_Co_) nanoparticles with *t*_Co_ = 0 nm (**a**), 0.05 nm (**b**), and 1 nm (**c**). Insets show the diagrams of size distribution for corresponding ensembles of nanoparticles (the units of abscissa axes are nanometers)

The size of the Fe/Co(*t*_Co_) сore/shell nanoparticles increases from ~ 6.3 to ~ 7.9 nm with the increase of calculated *t*_Fe_ from 0.05 to 2.5 nm (Fig. 4). Similarly to the case of Co/Fe(*t*_Fe_) nanoparticles, experimentally obtained shell thickness is smaller than the calculated one.

### Magnetic Measurements

**Fig. 5:**
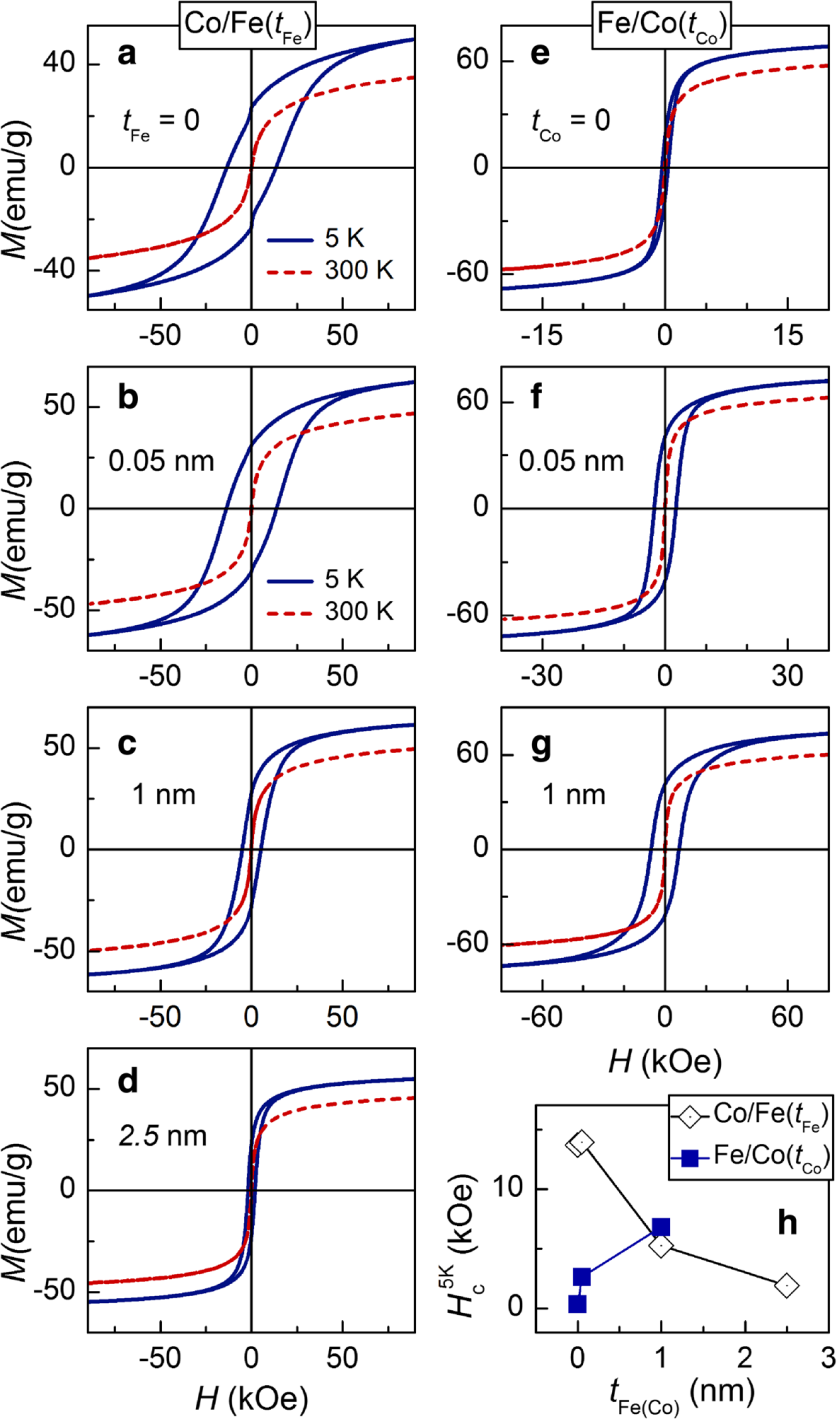
**a**–**g** Magnetic hysteresis loops *M*(*H*) for Co/Fe(*t*_Fe_) and Fe/Co(*t*_Co_) core/shell nanoparticles, measured at 5 and 300 K. **h** Dependence of coercivity measured at 5 K on the thickness of shell *t*_Fe(Co)_

Figure 5a–g shows magnetic hysteresis loops measured at 5 and 300 K for Co/Fe(*t*_Fe_) and Fe/Co(*t*_Co_) core/shell nanoparticles. It is seen that for both sets of samples, the addition of shell and subsequent increase in its thickness strongly affect the loop shape by modifying its parameters, in particular, saturation magnetization, *M*_s_, and coercivity, *H*_c_.

At 5 K, the values of the saturation magnetization for uncoated CoFe_2_O_4_ and Fe_3_O_4_ MNPs are equal to 50 and 77 emu/g, respectively. It is noteworthy that *M*_s_ equals 94 and 98 emu/g for respective bulk counterparts [21]. The reduced magnetization of the MNPs may result from a noticeable contribution from the near-surface layers which are usually characterized by the enhanced magnetic disorder. At the same time, one can conclude that the contribution to magnetization from the near-surface layers is higher in CoFe_2_O_4_ MNPs than in Fe_3_O_4_ ones.

Initial coating of MNPs (*t*_Fe(Co)_ = 0.05 nm) results in an increase of *M*_s_ for both sets of MNPs. At the same time, the growth of *M*_s_ is highly pronounced in Co/Fe(*t*_Fe_) samples and less expressed in Fe/Co(*t*_Co_) ones. This implies that the coating of MNPs strongly affects the properties of the near-surface layers of the core, at least for CoFe_2_O_4_ MNPs. For both sets of samples, the increase in the thickness of corresponding shells gives rise to a slight reduction of *M*_s_, as compared to the MNPs with the 0.05 nm shell. The rise of temperature to 300 K leads to a reduction of the saturation magnetization (by ~ 25% for Co/Fe(*t*_Fe_) MNPs and ~ 15% for Fe/Co(*t*_Co_) ones) but does not introduce qualitative changes in the *M*_s_ vs *t*_Fe(Co)_ behavior.

Dependence of coercivity measured at 5 K on the thickness of shells is shown in Fig. 5h. For Co/Fe(*t*_Fe_) MNPs, the initial coating of CoFe_2_O_4_ core with Fe_3_O_4_ shell (*t*_Fe_ = 0.05 nm) brings about only slight changes of *H*_c_—it remains near 13.8 kOe for both uncoated and coated MNPs. However, *H*_c_ sharply reduces with the further increase in *t*_Fe_—it drops to 5.27 kOe for *t*_Fe_ = 1 nm and reaches 1.93 kOe for *t*_Fe_ = 2.5 nm.

An opposite tendency is the characteristic of Fe/Co(*t*_Co_) MNPs; initial coating of Fe_3_O_4_ particles with CoFe_2_O_4_ shell (*t*_Co_ = 0.05 nm) results in the sharp increase of *H*_c_ from 0.38 to 2.65 kOe (almost one order of magnitude). As shell thickness rises further, the coercivity continues to increase and reaches 6.83 kOe for *t*_Co_ = 1 nm. This value is higher than *H*_c_ of Co/Fe(*t*_Fe_ = 1 nm). A reasonable explanation of the *H*_c_ vs *t*_Co_ dependence for Fe/Co(*t*_Co_) MNPs can be achieved assuming a simultaneous action of two factors—modification of the parameters of interfacial region between the core and shell, and contribution of magnetically hard shell to the enhancement of the total coercivity.

**Fig. 6:**
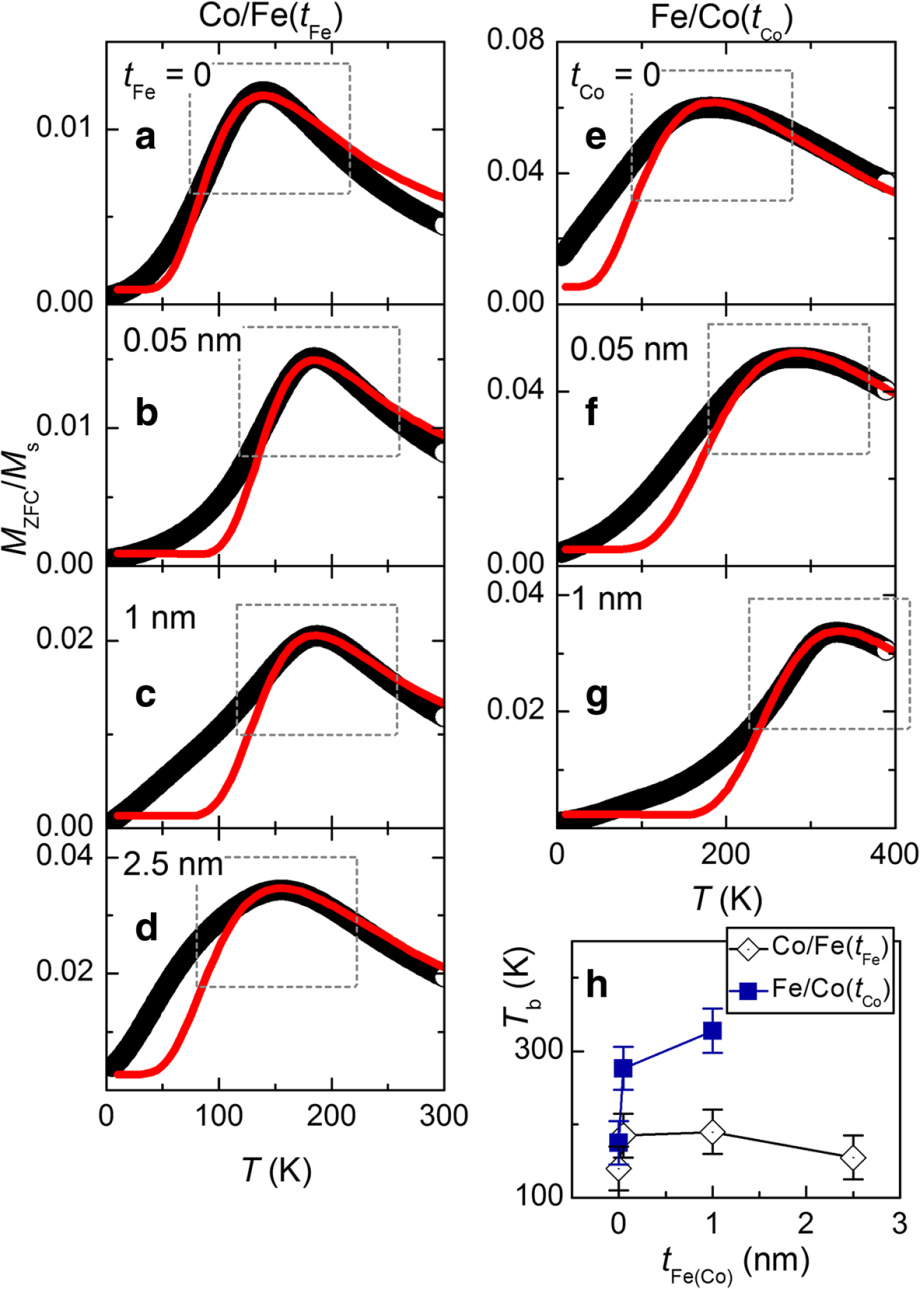
**a−g** Temperature dependences of normalized zero-field-cooled magnetization, *M*_zfc_(*T*)/*M*_s_, for Co/Fe(*t*_Fe_) and Fe/Co(*t*_Co_) MNPs: open circles—experimental data obtained in a field of 50 Oe; red solid lines—fitted curves with the use of Formula (2). Dotted rectangles show regions where the maximal correspondence between experimental and fitted curves was targeted. **h** Dependence of blocking temperature *T*_b_ on the thickness of shells

Temperature dependences of normalized zero-field-cooled magnetization, *M*_zfc_(*T*)/*M*_s_, for Co/Fe(*t*_Fe_) and Fe/Co(*t*_Co_) MNPs are shown in Fig. 6a–g. The data marked by circles were obtained experimentally in a field of 50 Oe. Each curve displays a maximum at a certain temperature *T*_b_ which is called blocking temperature. At this temperature, thermal energy becomes comparable to the anisotropy energy of MNPs, making the behavior of MNPs highly sensitive to external perturbations and conditions of the experiment. Below *T*_b_, the magnetic moments of the majority of particles are frozen on the time scale given by the experiment with their preferable orientations being governed by magnetic anisotropy. Above *T*_b_, the magnetic moments of the majority of particles can be considered as freely fluctuating, resulting in a superparamagnetic-like behavior of the ensemble.

The values of blocking temperature for uncoated CoFe_2_O_4_ and Fe_3_O_4_ MNPs are equal to 140 and 175 K, respectively. The reason for the fact that *T*_b_ for the CoFe_2_O_4_ MNPs is lower than that for the Fe_3_O_4_ ones is likely to originate from a smaller size of Co spinel nanoparticles.

Dependences of blocking temperature on the thickness of shells are shown in Fig. 6h. For both sets of MNPs, initial coating (*t*_Fe(Co)_ = 0.05 nm) leads to the rapid increase in *T*_b_. Further, the rise in the thickness of shells affects *T*_b_ not so strongly, as an initial coating. In our opinion, this fact additionally proves the idea that the primary effect of the MNP coating consists in the modification of the interfacial region between the core and shell.

The knowledge of *T*_b_ makes it possible to extract the information about characteristic features of the temperature dependence of coercivity. According to Reference [22], rough estimation of the changes of coercivity with temperature can be made using the formula:

1 $$ {H}_{\mathrm{c}}(T)={H}_{\mathrm{c}0}\left[1-{\left(T/{T}_{\mathrm{b}}\right)}^{0.5}\right] $$

where *H*_c0_ is the coercivity at *T* = 0 K. It follows from this formula that for all samples of Co/Fe(*t*_Fe_) set, coercivity becomes negligible at *T* > 200 K. On the other hand, for core/shell MNPs of the second set, *H*_c_ remains finite at *T* > 300 K, meaning that core/shell architecture is a powerful tool to tune coercivity of nanostructured magnetics.

## Discussion

To get a deeper insight into the processes governing the behavior of the core/shell nanoferrites, more detailed analysis of the obtained data has been performed. A simple model of non-interacting single domain particles [1] has been used for the fit of experimental *M*_zfc_(*T*)/*M*_s_ dependences shown in Fig. 6. The population of MNPs (given by a volume distribution *f*(*V*)) is sharply divided into two groups at each temperature, depending on their particular size—the fraction in an ideal superparamagnetic state that corresponds to MNPs below a certain critical volume and those, above such limit, whose magnetic moment remains blocked [23]:

2 $$ \frac{M_{\mathrm{ZFC}}}{M_{\mathrm{s}}}=\left[\underset{0}{\overset{V\mathrm{c}}{\int }}L\left({M}_{\mathrm{s}} HV/{k}_{\mathrm{B}}T\right)V\cdot f(V) dV+\underset{V\mathrm{c}}{\overset{\infty }{\int }}\left({M}_{\mathrm{s}}H/3{K}_{\mathrm{eff}}\right)V\cdot f(V) dV\right]/\underset{0}{\overset{\infty }{\int }}V\cdot f(V) dV, $$

where *L* is the Langevin function, *k*_B_ is the Boltzmann constant, *f*(*V*) is the volume distribution function, and *K*_eff_ is the particle effective anisotropy. In the first term, the low energy barrier approximation is used, where the energy barrier (defined as *K*_eff_*V*) is much smaller than the thermal energy *k*_B_*T*, and so can be neglected. Accordingly, the response of the magnetization to changes of magnetic field or temperature (*H* or *T*) follows a Langevin function. The second term component results from the initial susceptibility of randomly oriented single domain nanoparticles with effective anisotropy *K*_eff_. The threshold between the two populations is given by a critical volume *V*_c_:

3 $$ {V}_{\mathrm{c}}=\frac{k_{\mathrm{B}}T}{K_{\mathrm{eff}}}\ln \left(\frac{\tau_{\mathrm{m}}}{\tau_0}\right), $$

where *τ*_m_ is the characteristic measurement time, *τ*_0_ = 10^−9^ s [24, 25]. For quasistatic measurements, *τ*_m_ was chosen equal to100 s.

The results of the calculations are shown in Fig. 6a–g by red solid lines. In the process of the fitting, the lognormal distribution of MNPs in size was chosen, in compliance with the TEM data (see Figs. 3 and 4). The mode particle size *d*_σ_, at which a global maximum on the probability density function is achieved, was taken from TEM data and kept fixed. The width of the size distribution (standard deviation) and the value of *K*_eff_ were varied to reach maximal correspondence between experimental and fitted data. In the first place, the region in the vicinity of *T*_b_ was targeted (shown by dotted rectangles in Fig. 6a–g).

**Fig. 7:**
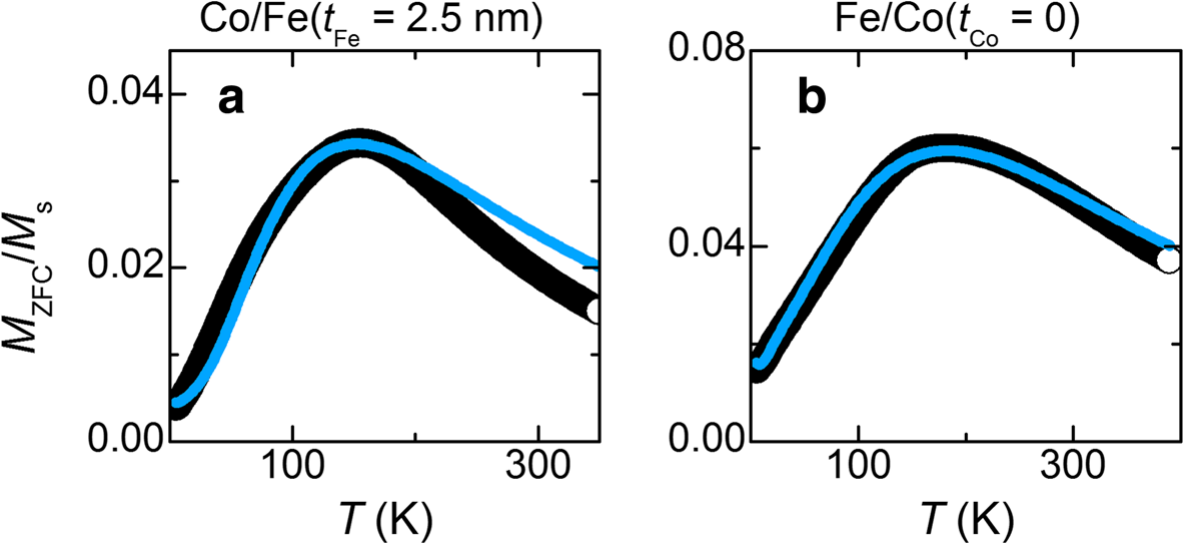
**a**, **b** Comparison of experimental *M*_zfc_(*T*)/*M*_s_ curves with simulated ones where the calculations were carried out with taking into account the presence of dispersion in *K*_eff_: (**a**) Co/Fe(*t*_Fe_ = 2.5 nm) sample; (**b**) Fe/Co(*t*_Co_ = 0) sample

The overall degree of correspondence between the experimental and fitted curves may be improved by taking into account the presence of dispersion not only in MNP size but also in other parameters. As an example, Fig. 7 demonstrates that almost ideal correspondence can be achieved by introducing a normal (Gaussian) distribution in *K*_eff_ (standard deviation is near 20% of *K*_eff_^max^). However, further analysis shows that *K*_eff_^max^ resulted from such calculations turns out to be equal to the anisotropy constant determined under the neglect of *K*_eff_ dispersion. Also, the results of such calculations do not add any important information to the discussion below. For this reason, the dispersion of *K*_eff_ was not accounted for in the remaining part of the paper.

The parameters resulted from the fitting procedure are collected in Table 1. The width of the size distribution, *σ*_d_, resulted from the fitting, turns out to be close to that obtained experimentally from TEM data (the difference does not exceed 10%). The anisotropy constant *K*_eff_ tends to be reduced in Co/Fe(*t*_Fe_) MNPs and increased in Fe/Co(*t*_Co_) ones, as the thickness of the corresponding shell grows. Such *K*_eff_ behavior is believed to be related to a redistribution of contributions to resulting MNP anisotropy from highly anisotropic Co ferrite and weakly anisotropic Fe_3_O_4_.

**Table 1:** Parameters used for calculations of *M*_zfc_(*T*)/*M*_s_ curves presented in Fig. 6a–g

*t*_Fe(Co)_, nm	0	0.05	1	2.5
*K*_eff_, × 10^6^ erg/cm^3^				
Co/Fe(*t*_Fe_)	9.7	11.2	7.5	1.9
Fe/Co(*t*_Co_)	1.8	4.5	5.8	–
*d*_σ_^a^, nm				
Co/Fe(*t*_Fe_)	4.1	4.2	5.0	7.3
Fe/Co(*t*_Co_)	6.3	6.4	7.9	–
*σ*_d_^b^, % of *d*_σ_				
Co/Fe(*t*_Fe_)	30	28	24	17
Fe/Co(*t*_Co_)	16	18	18	–

^a^The mode particle size *d*_σ_ which was taken from TEM data

^b^The width of lognormal distribution (standard deviation), as obtained from the fitting procedure

**Fig. 8:**
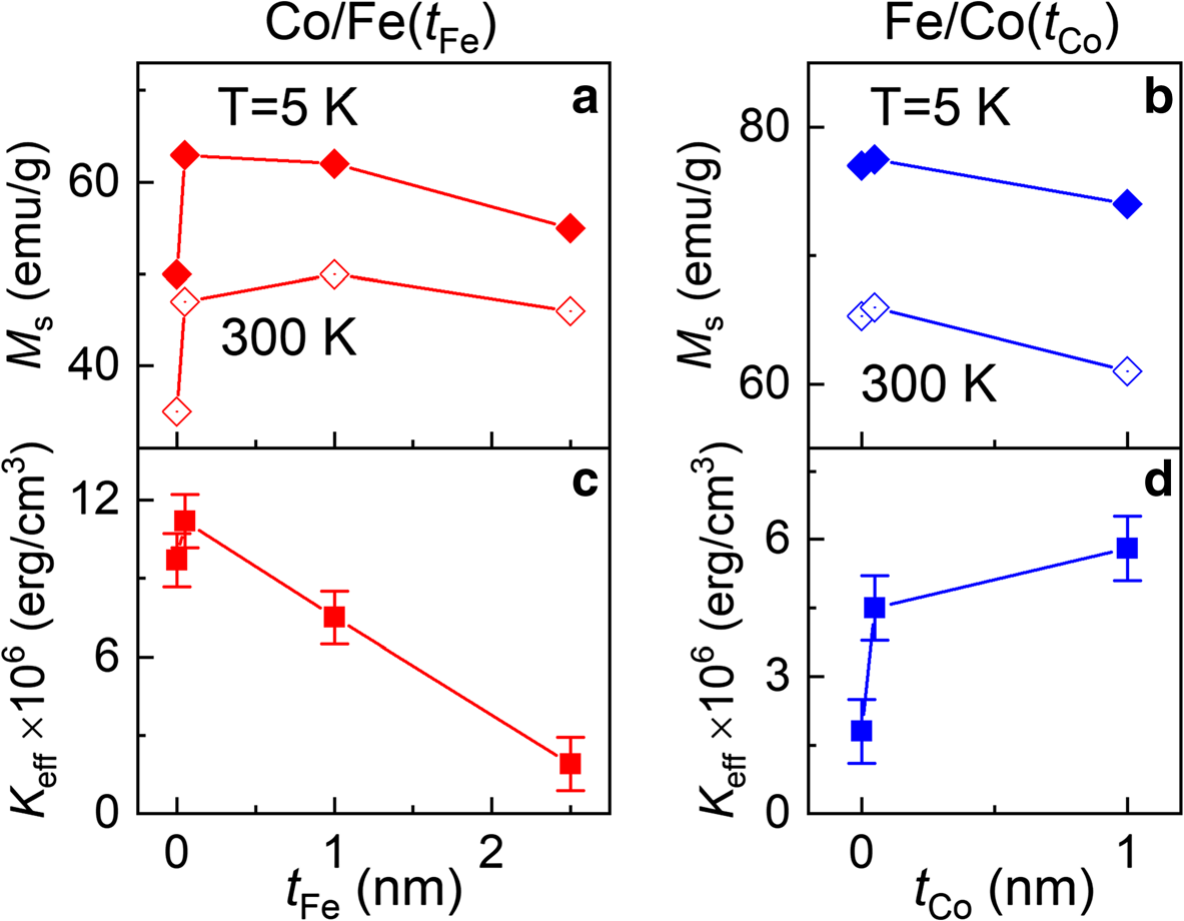
**a**–**d** Shell thickness dependences of saturation magnetization (**a**,**b**) and anisotropy constant (**c**,**d**) for Co/Fe(*t*_Fe_) (**a**,**c**) and Fe/Co(*t*_Co_) (**b**,**d**) MNPs

Figure 8 shows shell thickness dependences of saturation magnetization and anisotropy constant for Co/Fe(*t*_Fe_) and Fe/Co(*t*_Co_) MNPs. It is seen that with the use of core/shell architecture, it is possible to change principal magnetic parameters, *M*_s_ and *K*_eff_, over a wide range of their values. Two striking features of the graphs of Fig. 8 should be noted. First, an initial coating of MNPs with the shells may lead to rapid changes of the magnetic parameters of MNPs, which is especially expressed in Fig. 8a, d. This implies that one important effect from the addition of a shell is a modification of the parameters of the core-shell interface. Second, core/shell nanoparticles contain combined features of both the core and the shell (i.e., addition of shell with high anisotropy results in the increase of total anisotropy), but the resulting combination is not a simple summation of corresponding characteristics.

## Conclusions

Two sets of core/shell MNPs, CoFe_2_O_4_/Fe_3_O_4_ and Fe_3_O_4_/CoFe_2_O_4_, with varied thickness of shells were synthesized from metal chlorides in DEG solution. Single-phase spinel structural type for all samples was confirmed by XRD studies.

It is shown that for both sets of MNPs, the addition of shell strongly affects the shape of hysteresis loop and temperature dependences of magnetization. Based on a simple approach of coexistent superparamagnetic and blocked MNPs, the effective anisotropy constants were calculated. It is shown that in addition to the control of saturation magnetization, the use of core/shell architecture makes it possible to control the total effective anisotropy constant over a wide range of values.

It is concluded that coating of MNPs with the shells results in two simultaneous effects: first, it modifies the parameters of the core-shell interface, and second, it makes the particles acquire combined features of the core and the shell. The first effect becomes especially prominent when the parameters of core and shell strongly differ from each other.

## Abbreviations

DEG: Diethylene glycol
FC: Field-cooled
MNP: Magnetic nanoparticles
TEM: Transmission electron microscopy
XRD: X-ray diffraction
ZFC: Zero-field-cooled

## References

[1] Zhang Q, Castellanos-Rubio I, Munshi R (2015). Model driven optimization of magnetic anisotropy of exchange-coupled core-shell ferrite nanoparticles for maximal hysteretic loss. Chem Mater.

[2] Cho N-H, Cheong T-C, Min JH (2011). A multifunctional core–shell nanoparticle for dendritic cell-based cancer immunotherapy. Nat Nanotechnol.

[3] Mélinon P, Begin-Colin S, Duvail JL (2014). Engineered inorganic core/shell nanoparticles. Phys Rep.

[4] Noh SH, Na W, Jang JT (2012). Nanoscale magnetism control via surface and exchange anisotropy for optimized ferrimagnetic hysteresis. Nano Lett.

[5] Sattar AA, EL-Sayed HM, ALsuqia I (2015). Structural and magnetic properties of CoFe_2_O_4_/NiFe_2_O_4_ core/shell nanocomposite prepared by the hydrothermal method. J Magn Magn Mater.

[6] Song Q, Zhang ZJ (2012). Controlled synthesis and magnetic properties of bimagnetic spinel ferrite CoFe_2_O_4_ and MnFe_2_O_4_ nanocrystals with core–shell architecture. J Am Chem Soc.

[7] Lee J-H, Jang J-T, Choi J-S (2011). Exchange-coupled magnetic nanoparticles for efficient heat induction. Nat Nanotechnol.

[8] Ghosh R, Pradhan L, Devi YP (2011). Induction heating studies of Fe_3_O_4_ magnetic nanoparticles capped with oleic acid and polyethylene glycol for hyperthermia. J Mater Chem.

[9] Habib AH, Ondeck CL, Chaudhary P (2008). Evaluation of iron-cobalt/ferrite core-shell nanoparticles for cancer thermotherapy. J Appl Phys.

[10] López-Ortega A, Estrader M, Salazar-Alvarez G (2015). Applications of exchange coupled bi-magnetic hard/soft and soft/hard magnetic core/shell nanoparticles. Phys Rep.

[11] Belemuk AM, Chui ST (2013). Finite temperature performance of hard-soft composite nanomagnets and its dependence on geometry structure of composites. J Appl Phys.

[12] Akbari H, Sebt SA, Arabi H (2012). FePt_3_/CoFe_2_O_4_ core/shell nanostructures and their magnetic properties. Chem Phys Lett.

[13] El-Okr MM, Salem MA, Salim MS (2011). Synthesis of cobalt ferrite nano-particles and their magnetic characterization. J Magn Magn Mater.

[14] Maaz K, Mumtaz A, Hasanain SK, Ceylan A (2007). Synthesis and magnetic properties of cobalt ferrite (CoFe_2_O_4_) nanoparticles prepared by wet chemical route. J Magn Magn Mater.

[15] Iida H, Takayanagi K, Nakanishi T, Osaka T (2007). Synthesis of Fe_3_O_4_ nanoparticles with various sizes and magnetic properties by controlled hydrolysis. J Colloid Interface Sci.

[16] Feldmann C (2003). Polyol-mediated synthesis of nanoscale functional materials. Adv Funct Mater.

[17] Goloverda G, Jackson B, Kidd C, Kolesnichenko V (2009). Synthesis of ultrasmall magnetic iron oxide nanoparticles and study of their colloid and surface chemistry. J Magn Magn Mater.

[18] Yelenich OV, Solopan SO, Greneche JM, Belous AG (2015). Synthesis and properties MFe_2_O_4_ (M = Fe, Co) nanoparticles and core–shell structures. Solid State Sci.

[19] Peddis D, Orrù F, Ardu A (2012). Interparticle interactions and magnetic anisotropy in cobalt ferrite nanoparticles: influence of molecular coating. Chem Mater.

[20] Leung GW, Vickers ME, Yu R, Blamire MG (2008). Epitaxial growth of Fe_3_O_4_ (111) on SrTiO_3_ (001) substrates. J Cryst Growth.

[21] Chikazumi S, Graham, Jr, CD (1997) Physics of Ferromagnetism, Second Edition. Oxford University Press Inc, New York, ISBN 0-19-851776-9. https://books.google.com.ua/books?id=AZVfuxXF2GsC&printsec=frontcover&hl=uk&source=gbs_ge_summary_r&cad=0#v=onepage&q&f=false.

[22] Maaz K, Mumtaz A, Hasanain SK, Bertino MF (2010). Temperature dependent coercivity and magnetization of nickel ferrite nanoparticles. J Magn Magn Mater.

[23] Antoniak C, Farle M (2007). Magnetism at the nanoscale: the case of FePt. Mod Phys Lett B.

[24] Yelenich O, Solopan S, Kolodiazhnyi T et al (2015) Magnetic properties and AC losses in AFe_2_O_4_ (A = Mn, Co, Ni, Zn) nanoparticles synthesized from nonaqueous solution. J Chem 2015. 10.1155/2015/532198

[25] Yelenich OV, Solopan SO, Kolodiazhnyi TV et al (2013) Superparamagnetic behavior and AC-losses in NiFe_2_O_4_ nanoparticles. Solid State Sci 20. 10.1016/j.solidstatesciences.2013.03.013

